# Supplementary material for: Retroelement-Linked H3K4me1 Histone Tags Uncover Regulatory Evolution Trends of Gene Enhancers and Feature Quickly Evolving Molecular Processes in Human Physiology
Source: Cells. 2019 Oct 8;8(10):1219. doi: 10.3390/cells8101219 (PMC6830109; doi:10.3390/cells8101219)
Supplement: Supplementary file 1 [file cells-08-01219-s001.zip › cells-590484-SI/supplementary/Supplementary2.docx]

**Abbreviations**

RE - retroelement

TSS – gene transcription start site

1. **Calculation of RE-linked regulatory impact at the level of individual genes**
   1. *Gene RE-linked regulatory elements Enrichment score* (*GRE* score*).* To calculate the enrichment score for the RE-linked regulatory elements of a gene compared to averaged other genes, a value termed *Gene RE-linked Regulatory Elements Enrichment score*, or *GRE score* was introduced. Conceptually, *GRE* for an individual gene is the sum of RE-specific regulatory elements mapped in a 10kb-neighborhood of its TSS, that is normalized on the average content of RE-specific regulatory elements for all genes under investigation. For every individual gene, GRE score is calculated according to the formula:

$$GREg=\frac{NRg}{\frac{1}{n}\sum_{i=1}^{n}{NR}_{i}}$$

where *GRE_g_* is *GRE* score for a gene *g*; *NR_g_* is number of filtered and normalized over control ChIP-seq reads mapped on REs for a gene *g*; *i* is gene index and *NR_i_* is number of filtered and normalized over control ChIP-seq reads mapped on REs for a gene *i*; *n* is the total number of genes under investigation*.*

For every gene, the GRE score makes it possible to measure the extent of enrichment by the RE-linked regulatory elements. For example, GRE=1 means average impact on the regulation of a gene. GRE>1 means that the individual gene is enriched in RE-specific regulatory elements. Contrarily, GRE<1means that the gene has lower than average amount of RE-specific regulation .

- 1. *Normalized Gene RE-linked Regulatory Elements Enrichment score* *(NGRE* score*).* This value is introduced to calculate the enrichment of normalized RE-linked hits over the total normalized number of hits for a gene under investigation. Different genes have different regulation mechanisms and may have very different number of regulatory elements (both RE-linked and not) in their TSS neighborhood. The previous value (GRE score) can only give information if a gene is enriched by RE-linked regulatory elements relatively to other genes, but the same gene may be also enriched in total regulatory elements. It is important, therefore, to have a double normalized value showing if gene regulation is specifically enriched in RE-linked transcriptional regulation relatively to its total regulatory elements .

To define such RE-specific enrichment for an individual gene, a relative value termed *NGRE* was introduced for a gene *g*:

*NGRE_g_* = *GRE_g_*/*GTE_g_*

Here GRE value is as described in section 1.1. and *GTE* (*Gene Total regulatory Enrichment*) value characterizes gene-specific total regulatory elements distribution trends, expressed by the formula:

$$GTEg=\frac{TRNg}{TRNm}$$

where TRN_g_ is the total number of filtered and normalized over control ChIP-seq reads mapped in the 10-kb neighborhood of a gene *g* and *TRN_m_* is the mean TRN for all genes under investigation*.*

Higher *NGRE* value means higher impact of RE-specific regulation of a gene under investigation, and vice versa.

1. **Calculation of RE-linked regulatory impact at the level of molecular pathways**
   1. *Pathway Involvement Index* (*PII*). To assess the total impact of RE-linked regulatory elements on the regulation of an individual molecular pathway, a quantitative value was introduced termed *Pathway Involvement Index* (*PII*):

 $PIIp=\frac{\sum_{i=1}^{n} GREi}{n}$

where *PII_p_* is the PII score for a pathway *p*; *GRE_i_* is the *GRE* score for a gene *i* calculated according to section 1.1; *n* is the total number of genes in a pathway *p*.

To avoid misleading higher PII values for bigger pathways, *PII_p_* value is normalized on the number of genes in a pathway.

The bigger PII score suggest the higher impact of RE-linked regulatory elements on the overall regulation of a molecular pathway, and vice versa. However, PII score is not informative to assess the importance of RE-linked regulation of a pathway in the context of its total transcriptional regulation. To this end, the following metric was proposed.

- 1. *Normalized Pathway Involvement Index* (*NPII*). The Normalized Pathway Involvement Index (*NPII*) was introduced to estimate the relative RE-linked impact in the regulation of a molecular pathway:

*NPII_p_* = *PII_p_*/*PGI_p_*,

where *PII_p_* is a PII for a pathway *p* calculated according to 2.1;

*PGI_p_* is the *Pathway Gene-based Index* for a pathway *p* introduced to assess the impact of total regulatory elements (not only RE-linked) on the regulation of molecular pathways. *PGI* for a pathway *p* is expressed by the formula:

$$PGIp=\frac{\sum_{i=1}^{n} GTEi}{n}$$

where *GTE_i_* is the *GTE* score for a gene *i* calculated according to 1.2; *n* is the number of genes in a pathway *p*.

Higher *NPII* indicates higher *relative* impact of RE-linked regulatory elements in the total transcriptional regulation of a molecular pathway, and vice versa.
