# Supplementary figures and images for: Retroelement-Linked H3K4me1 Histone Tags Uncover Regulatory Evolution Trends of Gene Enhancers and Feature Quickly Evolving Molecular Processes in Human Physiology
Source: Cells. 2019 Oct 8;8(10):1219. doi: 10.3390/cells8101219 (PMC6830109; doi:10.3390/cells8101219)

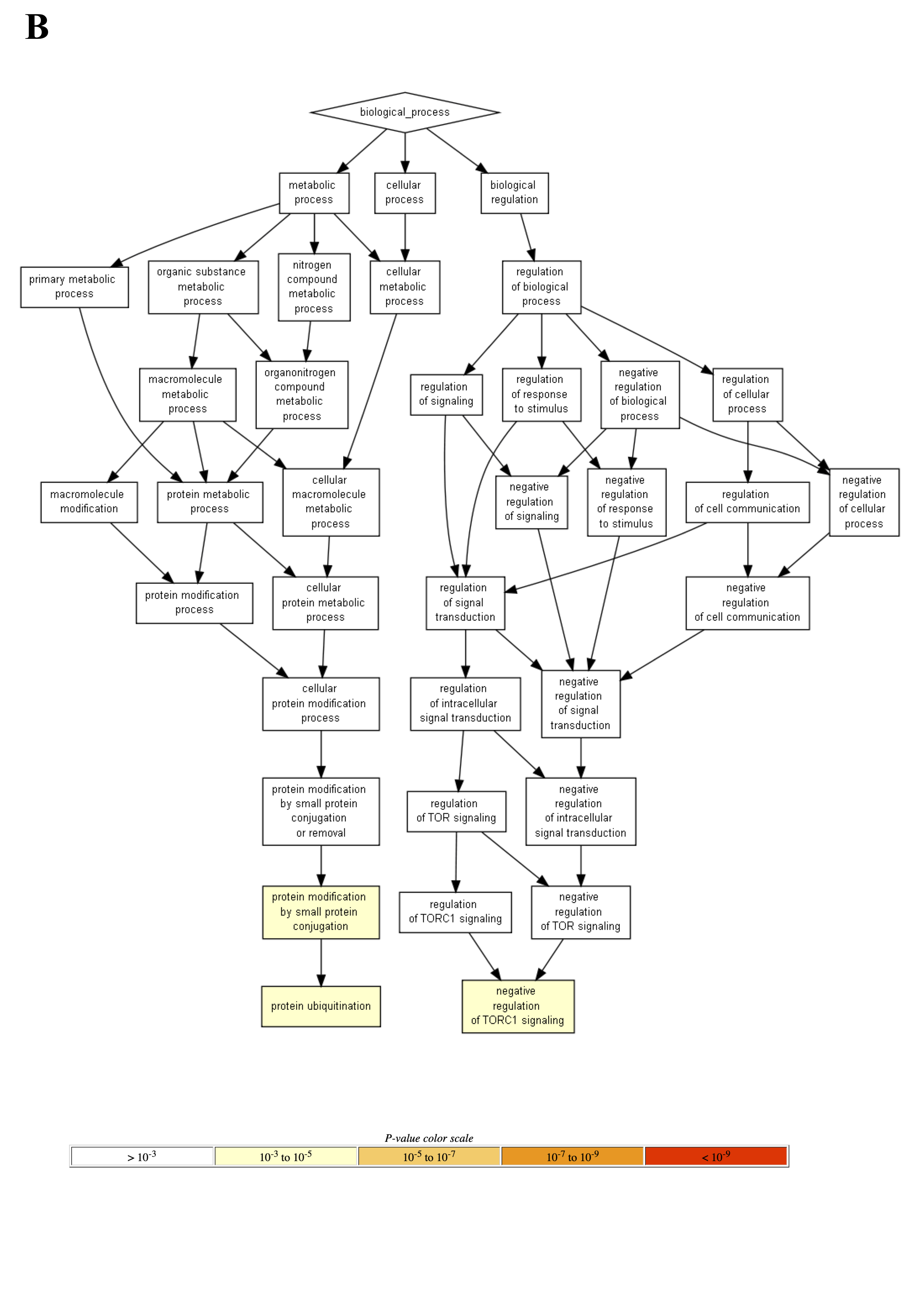

Supplement: Supplementary file 1 [file cells-08-01219-s001.zip › cells-590484-SI/supplementary/Supplementary10.jpg]

# Data analysis pipeline

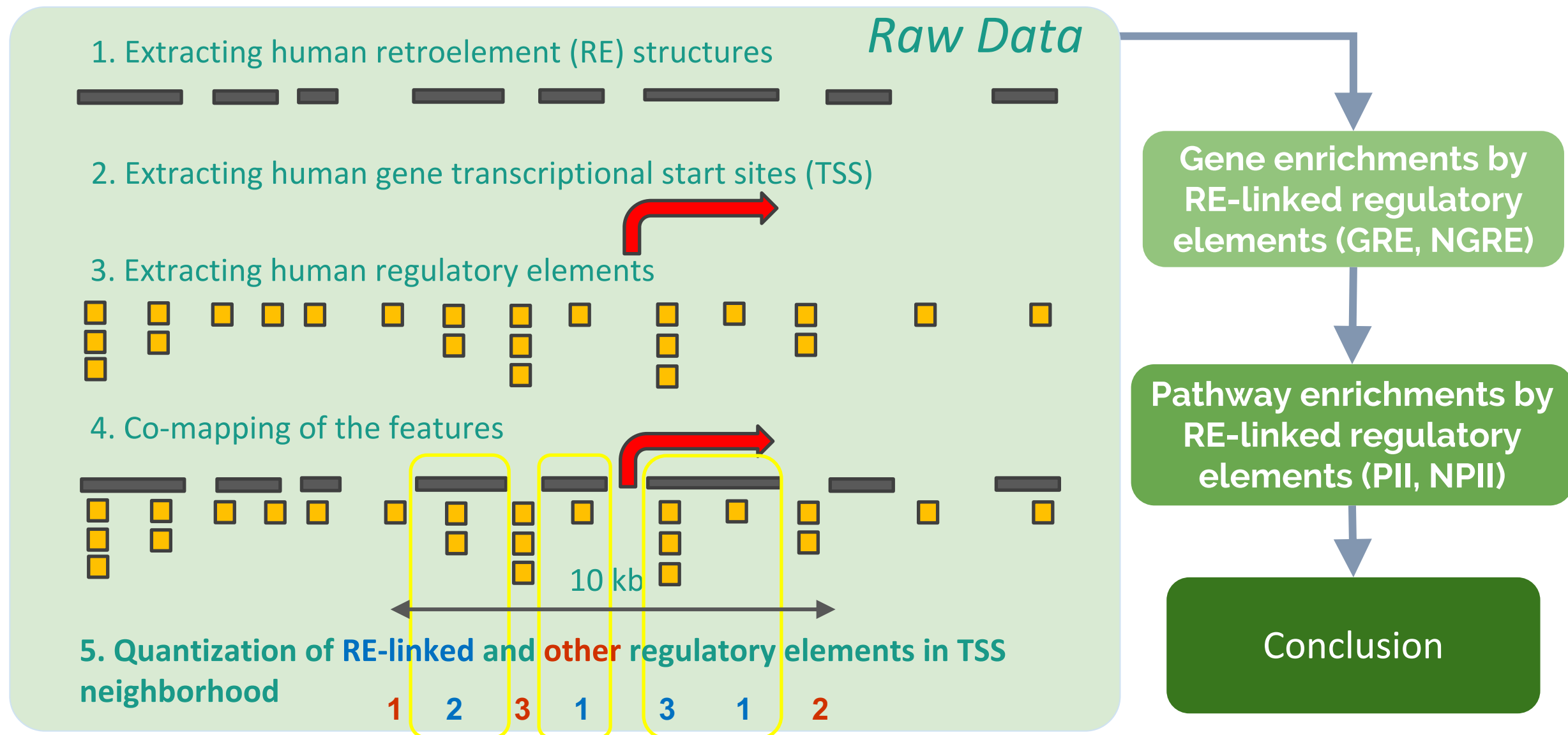

Supplement: Supplementary file 1 [file cells-08-01219-s001.zip › cells-590484-SI/supplementary/Supplementary3.pdf]

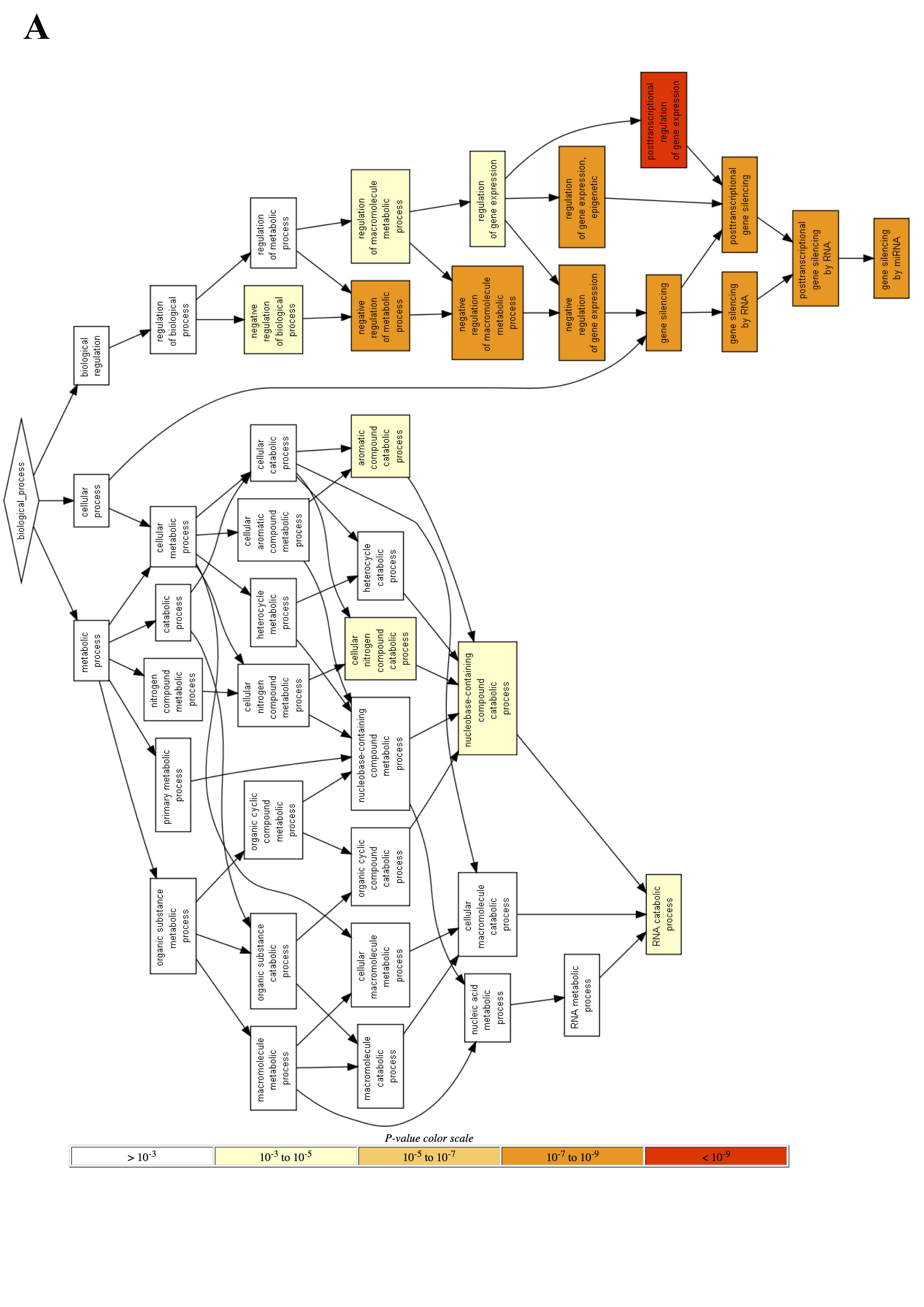

Supplement: Supplementary file 1 [file cells-08-01219-s001.zip › cells-590484-SI/supplementary/Supplementary9.jpg]
